# Sedentary behaviour and sleep quality

**DOI:** 10.1038/s41598-023-27882-z

**Published:** 2023-01-20

**Authors:** Mohammad Javad Koohsari, Akitomo Yasunaga, Gavin R. McCormack, Ai Shibata, Kaori Ishii, Yung Liao, Yukari Nagai, Koichiro Oka

**Affiliations:** 1 School of Knowledge Science, Japan Advanced Institute of Science and Technology, 1 Chome-1 Asahidai, Nomi, Ishikawa 923-1211 Japan; 2 Faculty of Sport Sciences, Waseda University, Tokorozawa, Japan; 3 Faculty of Liberal Arts and Sciences, Bunka Gakuen University, Tokyo, Japan; 4 Department of Community Health Sciences, Cumming School of Medicine, University of Calgary, Calgary, Canada; 5 Faculty of Kinesiology, University of Calgary, Calgary, Canada; 6 School of Architecture, Planning and Landscape, University of Calgary, Calgary, Canada; 7 Faculty of Health and Sport Sciences, University of Tsukuba, Tsukuba, Japan; 8 Graduate Institute of Sport, Leisure and Hospitality Management, National Taiwan Normal University, Taipei, Taiwan

**Keywords:** Public health, Epidemiology

## Abstract

High-quality sleep is an important factor in sustaining health and improving well-being. Previous evidence has demonstrated the positive associations between increased physical activity and reduced sedentary behaviour (SB) with sleep quality. The substitutional relationships between SB, light-intensity physical activity (LPA), and moderate-to-vigorous physical activity (MVPA) need to be considered when examining how a particular behaviour may impact sleep quality. No studies, to our knowledge, have explored these substitutional relationships in middle-aged adulthood. Using an isotemporal substitution approach, this study examined the associations of replacing sedentary time with physical activity on sleep quality measures in a sample of middle-aged adults in Japan. Data from 683 adults aged 40–64 living in Japan were used. The average daily time spent in SB, LPA, and MVPA was objectively assessed by accelerometers. Two self-reported sleep quality measures were obtained using questionnaires, including rest by sleep and sleep quality. Multivariable linear regression models were used to assess the associations of SB, LPA, and MVPA with the sleep quality measures stratified by gender. We found that each 60 min unit of SB or LPA replaced with MVPA was favourably associated with rest by sleep among women (*β* = 0.16, 95% CI 0.07, 0.28, *p* < 0.001; *β* = 0.18, 95% CI 0.07, 0.32, *p* < 0.05, respectively). There were no significant associations between SB, LPA, and MVPA with sleep measures in men across all three models. These findings indicate that higher MVPA has a positive association with sleep quality in middle-aged women.

## Introduction

High-quality sleep is an important factor in sustaining health and improving well-being^1^. Sleep quality is defined as “an individual’s self-satisfaction with all aspects of the sleep experience”^2^. A sleep disorder can be defined as “a disturbance in the quality and time of sleep”^3^. A systematic review and meta-analysis found that better sleep quality was favourably associated with various health outcomes such as cardiovascular disease^4^, metabolic syndrome^5^, mental health^6^, and dementia^7^. However, sleep disorders such as insomnia, narcolepsy, and excessive sleepiness, which have numerous adverse health effects, are prevalent in general populations worldwide^8^. For example, 50–70 million adults in the USA have a sleep disorder, with insomnia being the most common disorder^9^. In China, the pooled prevalence of insomnia was 15%, according to a meta-analysis of 17 studies^10^. Moreover, a gender difference in sleep quality has been reported in several studies^11–13^. In Japan, a nationwide study found that the prevalence of insomnia was higher in women (14.6%) relative to men (12.2%)^14^. Previous studies have reported that lifestyle habits such as a healthy diet and physical activity can improve sleep quality^15,16^.

Physical activity can have beneficial effects on sleep quality^17,18^. A systematic review revealed that moderate-intensity physical activity promotes adults’ sleep quality^17^. Notably, sedentary behaviour (SB) has also been linked to sleep quality, independent of physical activity levels^19^. SB is defined as "any waking behaviour characterised by an energy expenditure ≤ 1.5 metabolic equivalents (METs) while in a sitting or reclining posture”^20^. A systematic review and meta-analysis found that SB was associated with a higher risk of insomnia and sleep disturbance^19^. Another study using data from six countries found that sedentary time was associated with higher odds of sleep problems (e.g., poorer sleep quality), regardless of physical activity^21^. Interventions aimed at increasing physical activity and reducing SB may offer non-pharmacological strategies for improving sleep quality.

Nevertheless, there are methodological constraints in previous studies examining the associations between physical activity, sedentary time, and sleep quality. Since the total hours per day are stable, physical activity and sedentary time are interrelated daily activities, meaning that increasing one behaviour decreases the other behaviour. Therefore, these substitutional relationships between SB, light-intensity physical activity (LPA), and moderate-to-vigorous physical activity (MVPA) need to be considered when examining how a particular behaviour may impact sleep quality. To the best of our knowledge, however, only one previous study has examined the associations of reallocating time between sedentary time and different physical activity intensities on sleep outcomes among adults aged 55 years and older^22^. There is a lack of studies exploring these substitutional relationships during middle-aged adulthood, a life stage when age-related health, including sleep quality, begins^23^.

Using an isotemporal substitution approach, this study estimated changes in sleep quality associated with the substitution of 60 min of SB with physical activity in a sample of middle-aged adults in Japan. Isotemporal substitution model was “created to study time substitution effects of one activity substituting for another”^24^.

## Results

After excluding those with missing variables, data from 683 participants were included in this study (417 women, 263 men). Table 1 shows the characteristics of the sample. The mean age was 52.2 years, 61.1% were female, 82.0% were employed, 64.1% had completed advanced or higher education, 80.2% were a couple, 89.5% lived with others, and 55.1% had an annual gross household income over ¥5,000,000. The mean body mass index was 22.4 kg/m^2^, and 14.2% of the sample were smokers. On average, participants wore accelerometers for 15.4 h/per day. Participants spent an average of 8.3, 5.8, and 1.2 h/day in SB, LPA, and MVPA, respectively. Table 2 shows the bivariate correlation coefficients between accelerometer daily wear time, SB, LPA, and MVPA. There were significant correlations between SB and LPA (r = − 0.67), SB and MVPA (r = − 0.47), and LPA and MVPA (r = 0.27). Accelerometer daily wear time was also correlated with SB (r = 31), LPA (r = 0.44), and MVPA (r = 0.13).

**Table 1:** Characteristics of study participants (N = 683).

Variable	Mean (SD) or N (%)	Women (n = 417)	Men (n = 266)	*p*^*a*^
Age (years)	52.2 (7.1)	52.1 (6.9)	52.5 (7.4)	0.4
Body mass index	22.4 (3.2)	21.4 (2.9)	23.9 (3.1)	< 0.001
Working status				
*Employed*	560 (82.0)	311 (74.6)	249 (93.6)	< 0.001
*Unemployed*	123 (18.0)	106 (25.4)	17 (6.4)	
Educational attainment				
*Advanced or higher*	438 (64.1)	265 (63.5)	173 (65.0)	0.7
*Below advanced*	245 (35.9)	152 (36.5)	93 (35.0)	
Marital status				
*Single*	135 (19.8)	98 (23.5)	37 (13.9)	< 0.05
*Couple*	548 (80.2)	319 (76.5)	229 (86.1)	
Living status				
*Alone*	72 (10.5)	45 (10.8)	27 (10.2)	0.8
*With others*	611 (89.5)	372 (89.2)	239 (89.8)	
Gross annual household income				
≤ *¥5,000,000*	307 (44.9)	200 (48.0)	107 (40.2)	< 0.05
> *¥5,000,000*	376 (55.1)	217 (52.0)	159 (59.8)	
Smoking				
*No*	586 (85.8)	382 (91.6)	204 (76.7)	< 0.001
*Yes*	97 (14.2)	35 (8.4)	62 (23.3)	
Accelerometer wearing time (min/day)	921.7 (91.2)	937.7 (86.0)	896.5 (93.4)	< 0.001
SB (min/day)	500.3 (120.8)	486.2 (109.8)	522.5 (133.5)	< 0.001
LPA (min/day)	350.7 (110.7)	381.6 (97.9)	302.4 (112.5)	< 0.001
MVPA (min/day)	70.6 (39.4)	70.0 (36.1)	71.6 (44.2)	0.6
Rest by sleep	3.0 (0.7)	2.9 (0.6)	3.0 (0.7)	0.055
Sleep quality	2.5 (0.9)	2.4 (0.9)	2.5 (0.9)	0.129

^a^Based on an independent t-test or Chi-squared test.

*SB* sedentary behaviour, *LPA* light-intensity physical activity, *MVPA* moderate-to-vigorous physical activity.

**Table 2:** Bivariate correlation coefficients between accelerometer daily wear time, SB, LPA, and MVPA.

	Accelerometer daily wear time	SB	LPA	MVPA
Accelerometer daily wear time^a^	Referent	0.31**	0.44**	0.13**
SB ^b^		Referent	− 0.67**	− 0.47**
LPA^c^			Referent	0.27**
MVPA^d^				Referent

**p < 0.001.

^a^The sum of SB, LPA and MVPA.

^b^Sedentary behaviour.

^c^Light-intensity physical activity.

^d^Moderate-to-vigorous physical activity.

The results of regression analyses are shown in Tables 3 and 4. Each 60 min unit of SB or LPA replaced with MVPA was significantly and favourably associated with rest by sleep among women (*β* = 0.16, 95% CI 0.07, 0.28, *p* < 0.001; *β* = 0.18, 95% CI 0.07, 0.32, *p* < 0.05, respectively). Among men, there were no significant associations between SB, LPA, and MVPA with sleep measures in all three models.

**Table 3:** Isotemporal models examining the associations of SB, LPA, and MVPA with sleep measures in women (n = 417).

	SB	LPA	MVPA
	β (96%CI)	β (96%CI)	β (96%CI)
*Rest by sleep*			
Isotemporal			
Replacing SB with	Drop	− 0.05 (− 0.07, 0.03)	0.16 (0.07, 0.28)**
Replacing LPA with	0.06 (− 0.03, 0.067)	Drop	0.18 (0.07, 0.32)*
Replacing MVPA with	− 0.50 (− 0.28, − 0.07)**	− 0.50 (− 0.32, − 0.07)*	Drop
*Sleep quality*			
Isotemporal			
Replacing SB with	Drop	0.09 (− 0.02, 0.11)	0.09 (− 0.01, 0.28)
Replacing LPA with	− 0.10 (− 0.11, 0.02)	Drop	0.06 (− 0.09, 0.26)
Replacing MVPA with	− 0.28 (− 0.28, 0.01)	− 0.16 (− 0.26, 0.09)	Drop

Regression coefficients correspond to a 60 min/day increment of each activity.

All models were adjusted for age, education, marital status, body mass index, living status, income, working status, and smoking. The single-activity and isotemporal models were also adjusted for the accelerometer daily wear time.

*SB* sedentary behaviour, *LPA* light-intensity physical activity, *MVPA* moderate-to-vigorous physical activity.

*p < 0.05; **p < 0.001.

**Table 4:** Isotemporal models examining the associations of SB, LPA, and MVPA with sleep measures in men (n = 263).

	SB	LPA	MVPA
	β (96%CI)	β (96%CI)	β (96%CI)
*Rest by sleep*			
Isotemporal			
Replacing SB with	Drop	− 0.02 (− 0.06, 0.05)	0.03 (− 0.09, 0.15)
Replacing LPA with	0.03 (− 0.05, 0.06)	Drop	0.04 (− 0.11, 0.18)
Replacing MVPA with	− 0.10 (− 0.15, 0.09)	− 0.11 (− 0.18, 0.11)	Drop
*Sleep quality*			
Isotemporal			
Replacing SB with	Drop	− 0.03 (− 0.09, 0.06)	− 0.01 (− 0.17, 0.15)
Replacing LPA with	0.04 (− 0.06, 0.09)	Drop	0.00 (− 0.19, 0.20)
Replacing MVPA with	0.02 (− 0.15, 0.17)	− 0.01 (− 0.20, 0.19)	Drop

Regression coefficients correspond to a 60 min/day increment of each activity.

All models were adjusted for age, education, marital status, body mass index, living status, income, working status, and smoking. The single-activity and isotemporal models also were adjusted for the accelerometer daily wear time.

*SB* sedentary behaviour, *LPA* light-intensity physical activity, *MVPA* moderate-to-vigorous physical activity.

## Discussion

This is the first study, to our knowledge, to undertake an isotemporal substitution analysis to examine the hypothetical associations of replacing sedentary time with physical activity on sleep quality measures in a sample of middle-aged adults. In recent years, some researchers recommended using a newer compositional substitution model (compositional data analysis) in which movement behaviours are treated as relative values because it may be more appropriate to treat collinear behaviours such as sleep, SB, LPA, and MVPA as relative rather than absolute values^25,26^. However, Mekary et al.^27^ suggested that the absolute values of activities instead of relative values can be more meaningful in physical activity epidemiology for the following reasons: (a) physical activity guidelines are usually given in absolute amounts, and (b) “a certain percentage of total discretionary activity time could be very heterogeneous among individuals, which makes it hard to interpret and establish physical activity guidelines because different individuals often have widely different total discretionary time available for physical activities”^27^. A study by Biddle et al.^28^, which examined the association between activity and cardiometabolic health using both the isotemporal substitution model and compositional data analysis, also reported that results from the isotemporal substitution model and compositional data analysis were mainly identical in direction or magnitude. We used the isotemporal substitution model in this study because our paper aimed to identify how much sitting time may be replaced by physical activity to improve sleep quality. Several previous studies have shown that being physically active can improve sleep quality^17^. However, the intensity and amount of physical activity needed to influence sleep quality in middle-aged adults remain to be explored. Using the isotemporal substitution model, our study provided preliminary cross-sectional evidence on the positive associations of certain intensities and amounts of MVPA with sleep quality.

Our study found that replacing 60 min of SB or LPA with the same amount of MVPA was positively associated with the rest by sleep measure among women. We were unable to compare our findings with other studies since we did not come across any studies assessing the substitutional associations of sedentary time and physical activity with sleep quality among middle-aged adults. These findings align with a body of research on the positive associations of MVPA with sleep^17,18,29,30^. For instance, a systematic review and meta-analysis found that exercise was associated with better sleep quality^18^. MVPA may influence sleep quality by affecting several potential pathways, such as body temperature and metabolic and endocrine functions^31^. There were no significant differences in sleep quality measures between women and men. However, the associations were only significant among women. Sleep quality may have different correlates between women and men. In Canada, physical activity, SB, and sleep recommendations are combined into 24-h movement guidelines^32^. Our findings suggest that if adults (women) replace SB with MVPA, they may achieve better quality sleep which potentially could help them achieve the recommended levels of sleep suggested for optimal health. By addressing two parts of the recommendation (decrease SB and increase MVPA), achieving the third part of the guidelines (increased sleep) might be more likely.

No significant associations were observed in the associations of replacing SB with LPA on sleep quality measures in women and men. This is in contrast with a previous study conducted among older adults examining the substitutional associations of sedentary time and physical activity with sleep quality^33^. Seol et al.^33^ found that substituting 30 min of SB with LPA was associated with better older adults’ sleep quality measures such as sleep efficiency, awakenings, and sleep fragmentation index. The exact reasons for the observed null findings in our study in relation to LPA are yet to be known. The LPA in our sample may have consisted of ‘required’ household or workplace activities such as cooking, washing dishes, doing laundry, photocopying, and ironing. While these household or workplace activities may contribute to physical health, the compulsory nature of such activities (and lack of joy) may increase stress in some adults. Similarly, another study using isotemporal models found that replacing SB with LPA had no positive association with health-related quality of life in older adults^34^. Further research is needed to explore how different types of LPA may influence sleep quality in middle-aged adults.

This study had some limitations. Since this was a cross-sectional study, we were unable to determine causal relationships between SB, LPA, MVPA, and sleep variables. Because of the rather small sample size, the findings may not represent Japan's general middle-aged adults' population. While we adjusted the models for the working status and body mass index, these variables do not necessarily capture the nature of work (e.g., shift work, type of work) and obesity that may be associated with physical activity and sleep quality. Furthermore, similar to previous studies using accelerometers, we could not measure physical activities involving only upper or lower limbs without trunk movement and water-based activities. The sleep quality measure was a past month recall, whereas physical activity was based on a single week. Sleep quality was also self-reported, and no prior study reported their validity (although it can be assessed objectively, it is less convenient in larger samples because it usually requires a sleep study at a clinic). The sleep quality measures may not reflect all aspects of sleep quality, including sleep duration. The average vigorous physical activity was low in our sample, with about 78% of participants having only less than 1-min vigorous physical activity. Future studies are needed to identify the separate associations of vigorous physical activity and moderate physical activity with sleep quality. The strength of this study included objectively-measured physical activity and sedentary time, the focus on the middle-aged group, and the use of a novel isotemporal substitutional model.

## Conclusions

The present study was designed to explore the cross-sectional associations of replacing SB with LPA and MVPA on sleep quality among middle-aged adults. It was shown that the replacement of 60 min of SB with MVPA was associated with better rest by sleep among women. Therefore, it seems that MVPA has a beneficial association with sleep quality in middle-aged women in this sample. Further studies are necessary to confirm these findings in other populations.

## Methods

### Study participants and data collection

Participants were drawn from a larger epidemiological study conducted in two Japanese urban localities, Koto Ward and Matsuyama City in Japan. Detailed methods of study recruitment and sampling procedure have been documented elsewhere^35,36^. Briefly, to recruit the participants, an invitation letter was mailed to 6,000 middle-aged adult residents (aged 40–64 years), randomly sampled from the Japan government registry of residential addresses between July to December 2013 and April 2014 to February 2015. A reminder letter was posted to non-respondents two weeks after the initial mailing. A total of 866 individuals (response rate = 14.4%) took part in the study. A self-administered questionnaire and an accelerometer were mailed to each participant. A book voucher (¥1000, equivalent to about US$10) was offered to those participants who completed the study (n = 779). All participants provided written informed consent prior to participation. The study was approved by the Research Ethics Committee, Waseda University, Japan (2012–269) and the methods were carried out in accordance with these guidelines.

### Measures

#### Sleep quality measures

Two sleep quality measures, including rest by sleep and sleep quality, were assessed with two items used previously in the National Health and Nutrition Survey in Japan^37,38^. Participants responded to the following two questions: (a) In the past month, have you been able to get enough rest from sleep? (b) During the past month, have you had trouble falling asleep, waking up in the middle of the night, waking up early in the morning, or not sleeping well? Participants responded to these questions using a four-point Likert scale (very poor, poor, good, or very good, and often, sometimes, rarely, or not at all, respectively). Rest by sleep answers were reverse coded before the analysis; therefore, higher scores indicate better sleep quality for both measures.

#### Physical activity and sedentary behaviour

A validated triaxial accelerometer (Active style Pro, HJA-750C; Omron Healthcare, Kyoto, Japan) was used to objectively assess participants’ physical activity and sedentary time for seven consecutive days. The detailed data processing choices and validity of this accelerometer device have been reported elsewhere^39,40^. This accelerometer device assesses the intensity of activity by METs employing a built-in algorithm. The participants were instructed to wear the accelerometer on their waist for at least seven consecutive days except when sleeping or during water-based activities. Non-wear time was defined as at least intervals of at least 60 consecutive min of 0 count value per minute (cpm), with allowance for up to two minutes of observations of some limited movement (< 50 cpm) within these periods^39,41^. To be included in the study, participants needed to wear the accelerometer for ≥ 4 days (including one non-working day), with ≥ 10 h/day of wear time each day^42^. The daily average time spent in SB (≤ 1.5 METs), LPA (> 1.5 to < 3.0 METs) and MVPA (≥ 3.0 METs) were calculated^20,43^.

#### Covariates

Participants reported the following sociodemographic variables: age, gender (women, men), educational attainment (advanced or higher, below advanced), marital status (single, couple), living status (alone, with others), gross annual household income (≤ ¥5,000,000, > ¥5,000,000), working status (employed, unemployed), and smoking (yes, no). Body mass index was measured using participants’ reported weight and height. Accelerometer daily wear time, which is the sum of SB, LPA and MVPA, was also included as a covariate.

### Statistical analysis

Descriptive statistics were estimated for covariates, sedentary time and physical activity, and sleep quality variables. Independent t-tests and Pearson’s chi-square test were used to compare these variables between women and men. Multivariable linear regression models were used to assess the associations of SB, LPA, and MVPA with two sleep measures of rest by sleep and sleep quality stratified by gender. Isotemporal substitution model can be used to “estimate the effect of replacing one physical activity type with another physical activity type for the same amount of time”^24^. We used 60 min as a unit for activity; thus, the isotemporal models examined the association of replacing 60 min of one activity with the same amount of another activity. This time unit was chosen because this is the time recommended by the official Japanese physical activity guideline for middle-aged adults^44^.

The isotemporal model examined the association of substituting one activity type with another for an equal time. The isotemporal model (in the case of omitting SB from the model) is shown as follows:

$$\begin{aligned} {\text{Outcome variable}} & = \left( {{\text{b}}_{{1}} } \right){\text{LPA}} + \left( {{\text{b}}_{{2}} } \right){\text{MVPA}} + \left( {{\text{b}}_{{3}} } \right){\text{covariates accelerometer daily wear time}} \\ & \quad + \left( {{\text{b}}_{{4}} } \right){\text{covariates}}{.} \\ \end{aligned}$$

The coefficients b_1_ and b_2_, for instance, can be interpreted as the association of replacing SB with LPA or MVPA for 60 min while holding other activities and total wear time constant. A complete case approach was chosen because the proportion of missing data was low^45^. For all point estimates (*β* = standardised regression coefficients), 95% confidence intervals (CIs) were estimated. All inferential statistical tests were two-tailed and statistical significance was set at *p* < 0.05. Analysis was undertaken using IBM SPSS Statistics V.20.0 for Windows (IBM Japan).

## Data Availability

The datasets generated and/or analysed during the current study are not publicly available due to ethical and legal constraints but anonymized data are available from the corresponding author on reasonable request.
